# eCOMPAGT integrates mtDNA: import, validation and export of mitochondrial DNA profiles for population genetics, tumour dynamics and genotype-phenotype association studies

**DOI:** 10.1186/1471-2105-11-122

**Published:** 2010-03-09

**Authors:** Hansi Weißensteiner, Sebastian Schönherr, Günther Specht, Florian Kronenberg, Anita Brandstätter

**Affiliations:** 1 Division of Genetic Epidemiology, Department of Medical Genetics, Molecular and Clinical Pharmacology; Innsbruck Medical University, Innsbruck, Austria; 2 Department of Database and Information Systems; Institute of Computer Science, University of Innsbruck, Innsbruck, Austria

## Abstract

**Background:**

Mitochondrial DNA (mtDNA) is widely being used for population genetics, forensic DNA fingerprinting and clinical disease association studies. The recent past has uncovered severe problems with mtDNA genotyping, not only due to the genotyping method itself, but mainly to the post-lab transcription, storage and report of mtDNA genotypes.

**Description:**

eCOMPAGT, a system to store, administer and connect phenotype data to all kinds of genotype data is now enhanced by the possibility of storing mtDNA profiles and allowing their validation, linking to phenotypes and export as numerous formats. mtDNA profiles can be imported from different sequence evaluation programs, compared between evaluations and their haplogroup affiliations stored. Furthermore, eCOMPAGT has been improved in its sophisticated transparency (support of MySQL and Oracle), security aspects (by using database technology) and the option to import, manage and store genotypes derived from various genotyping methods (SNPlex, TaqMan, and STRs). It is a software solution designed for project management, laboratory work and the evaluation process all-in-one.

**Conclusions:**

The extended mtDNA version of eCOMPAGT was designed to enable error-free post-laboratory data handling of human mtDNA profiles. This software is suited for small to medium-sized human genetic, forensic and clinical genetic laboratories. The direct support of MySQL and the improved database security options render eCOMPAGT a powerful tool to build an automated workflow architecture for several genotyping methods. eCOMPAGT is freely available at http://dbis-informatik.uibk.ac.at/ecompagt.

## Background

Human mitochondrial DNA (mtDNA) is routinely analyzed in various disciplines, such as medical genetics (searching for inheritable or somatic pathogenic mutations), population genetics (studying the differences between human populations), and forensic genetics (estimating the chance of a random match between two samples).

The strict maternal inheritance of mtDNA results in a natural grouping of sequence haplotypes into monophyletic clusters, referred to as haplogroups [1]. The members of a haplogroup carry a specific sequence motif as a consequence of sharing a common ancestor. As a general practice, mtDNA haplotypes are reported as a list of nucleotide positions that deviate from the revised Cambridge Reference Sequence (rCRS; [2]).

Unfortunately, mtDNA sequencing efforts are quite often affected by technical and interpretation problems. Sample mix-up, contamination, biochemical problems, use of a wrong reference sequence and transcription errors lead to "phantom mutations" [3], which in turn result in false associations and erroneous conclusions [4-7]. Even forensic databases, which are used to estimate the rarity of an mtDNA profile, were hit by a variety of errors [8-10] and as a consequence, guidelines concerning mtDNA genotyping were issued [10-14]. These especially recommend double evaluation of mtDNA sequences, a check for phylogenetic consistency using haplogroup determination and the construction of networks for the detection of phantom mutations [15]. In addition, specific computer programs can automatically export mtDNA profiles as differences to the rCRS (e.g. Sequencher (GeneCodes, Ann Arbor, USA) or SeqScape (Applied Biosystems, Foster City, USA)).

We present a new and extended version of eCOMPAGT [16], a database system which now allows the successful data handling and quality assurance of mtDNA genotypes for medical genetics, population genetics and forensics. eCOMPAGT imports mtDNA profiles generated with Sequencher or SeqScape, compares double evaluations of profiles for validation, stores profiles in a relational database system and allows different export formats for disease-association studies, population genetics, forensic databases and for the construction of phylogenetic networks. In particular, the search for inherited pathogenic mutations is facilitated by linking mtDNA polymorphisms to phenotypes, thus enabling a hypothesis-free scan for new genotype-phenotype associations when all common mtDNA polymorphisms are covered. Furthermore, the study of somatic mtDNA instabilities caused by carcinogenesis or by the action of a particular toxin is enabled by the possibility to import mtDNA profiles from different tissues and the automatic export of alteration patterns. Finally, haplogroup affiliations of samples can also be stored, thus enabling a phylogenetic view of the mtDNA profiles.

## Construction and Content

**Figure 1:**
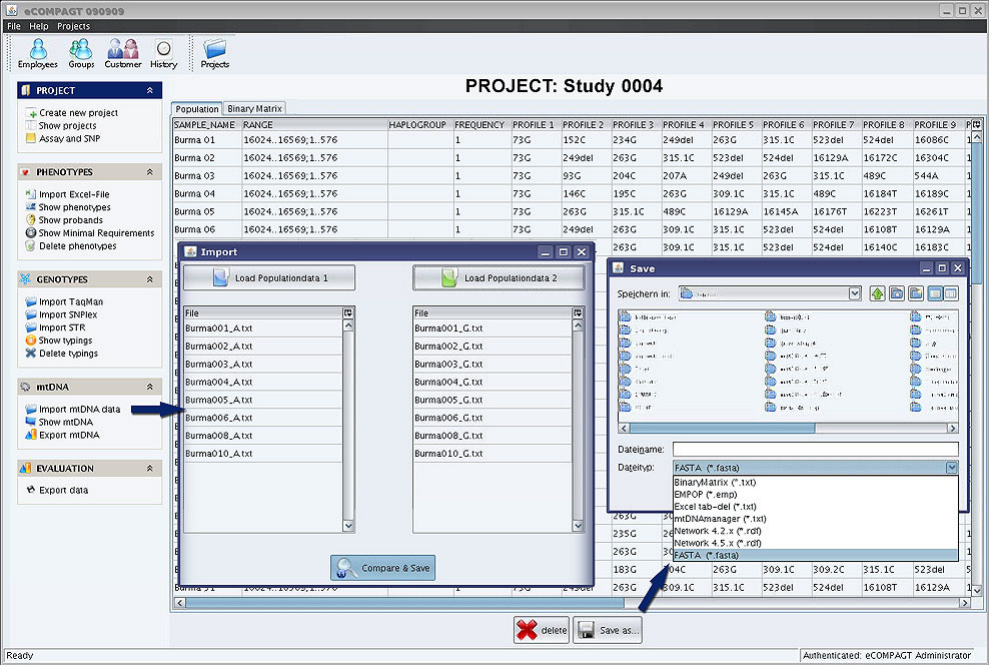
**eCOMPAGT's mtDNA GUI**. The new menu entry on the left side „mtDNA“ allows the management of mtDNA profiles. The menu item "Import" reads two lists of files and compares them pairwise, the menu item "Show mtDNA" gives an overview of the imported data and allows an export of data to different file formats. "Export mtDNA" enables the combination of phenotypes and mtDNA data.

eCOMPAGT [16] is a Laboratory Information Management System (LIMS) designed to store the complete set of project-relevant information in a compact, straightforward and efficient way. Previously published features of eCOMPAGT, such as the phenotype and genotype storage capacity, the user friendly export interface for the combination of genotypes and phenotypes, the versioning component for traceability and the widespread project and customer management have been optimized. eCOMPAGT has been further enhanced by the option to store and evaluate mitochondrial DNA (mtDNA) data in various ways (Figure 1).

The goal was to integrate the workflow of mtDNA analysis into eCOMPAGT to securely import, validate, store and export mtDNA profiles and to connect mtDNA polymorphisms with phenotypic data by making use of eCOMPAGT's combination and evaluation features. Errors in steps such as transcription of mtDNA profiles, merging of different samples into one file, converting data for phylogenetic programs or connecting genotypes to phenotypes for disease association studies are thus avoided. The newly developed mtDNA capacity allows the **import **of data from the alignment and analysis software systems SeqScape and Sequencher from two independent scientists in the form of mutation reports (examples of accepted mutation report formats can be found at http://dbis-informatik.uibk.ac.at/ecompagt). Furthermore, the import of mtDNA data can be performed for population data, gene-phenotype association or tumour studies (for the identification of somatic mutations in tumour tissue compared to benign cells), in which case the project type needs to be selected initially using eCOMPAGT's project management capability.

For quality management purposes, it is recommended that all mtDNA sequences are evaluated by two different scientists or lab technicians [17]. These two evaluations should be checked for consistency by a senior mtDNA expert, a process that is called "validation". eCOMPAGT enables the **validation **of sequence evaluations of mtDNA profiles from two different files derived from two different evaluators and from different sequence evaluation programs. eCOMPAGT generates reports which indicate differences between the evaluations for the senior validating scientist; it then stores the appropriate file - selected by the scientist - securely in a relational database. The imported data can be displayed as EMPOP (EDNAP mtDNA population database) format [17,18] or as a binary matrix, further supported by an **export **interface to generate different kinds of formats (EMPOP [17,18], binary matrix, tab-delimited files for Excel, mtDNAManager [19], Network.exe [20], and FASTA).

## Specific mtDNA features

### Import and validation of mtDNA

**Figure 2:**
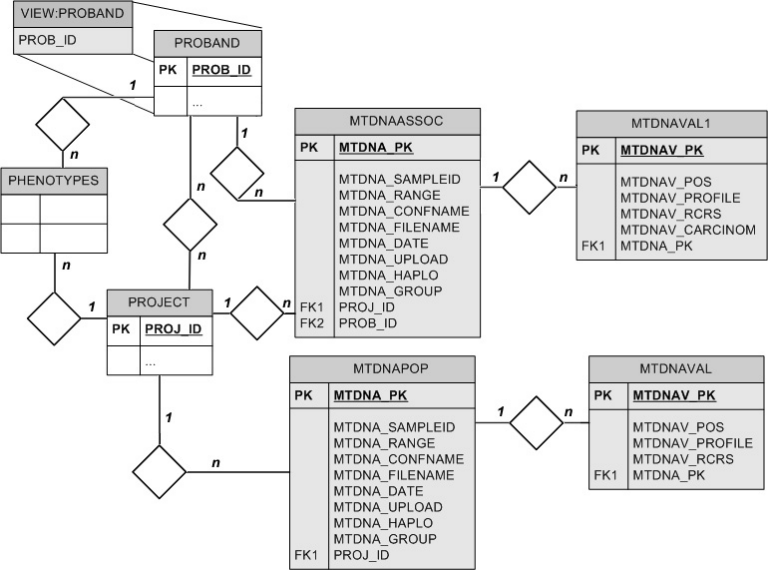
**Entity relationship diagram of the mtDNA extension of eCOMPAGT**. Four new relations are introduced: MTDNAASSOC, MTDNAPOP and the corresponding values MTDNAVAL/MTNDAVAL1. Entities are represented as rectangles and relationships as diamonds.

eCOMPAGT is constructed in a way that allows the import of different kinds of mtDNA project types (Figure 2). At project creation, the project type (population data, association study or tumour study) needs to be specified, which then automatically generates the corresponding graphical user interface (GUI).

The import interface supports the simultaneous upload of hundreds of mutation reports (Seqscape or Sequencher), including two mutation reports from two different scientists for each sample and performs a row by row (i.e. polymorphism by polymorphism) comparison of the different sequence evaluations for each sample. Human mtDNA variation is recorded by aligning mtDNA sequences to the rCRS. This task is straightforward for the vast majority of nucleotide positions, but is difficult in regions displaying length variation. Different alignments of the same sample can result in mismatches on several nucleotide positions. To remedy the situation, samples should be evaluated and aligned by at least two mtDNA analysts, and a third person should perform a comparison of these two evaluations. If differences occur between the two different reports from one sample, eCOMPAGT indicates those differences and allows the validating scientist to either select the appropriate file or to additionally import, upload and store a third sequence evaluation. If no profile differences occur, one of the identical files is stored in the database automatically. In addition, if the sequence mutation reports were generated with Sequencher, the sequenced range of the sample is also stored, as this information is exported by Sequencher. A short video describing this very important feature can be found at http://dbis-informatik.uibk.ac.at/ecompagt.

**Figure 3:**
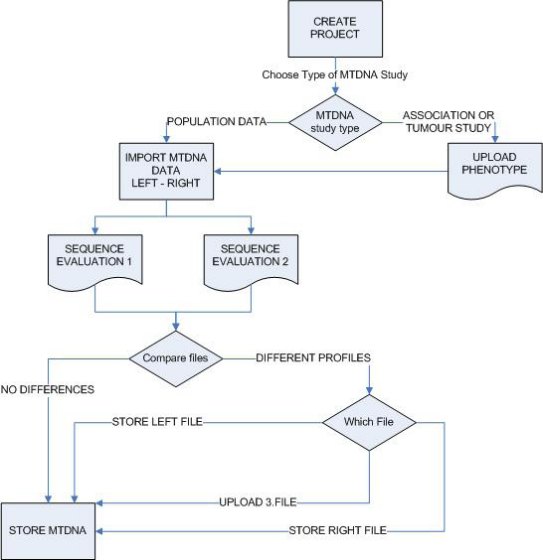
**Flowchart of the mtDNA uploading process**. mtDNA mutation reports can be uploaded from two different sequence evaluators per sample and from different sequence evaluation programs (SeqScape or Sequencher). The different sequence reports are automatically compared with each other; equal reports are stored automatically, while inconsistent reports can be revised by the validating senior scientist, who can also upload a third evaluation for final storage.

It is important to note that phenotypes can be easily imported at any time into an existing project, by specifying the relevant ID column at the time of upload. For genotypes we decided to use the filename of the mutation report as an identifier. More precisely, we are using the underscore ("_") as a separator to extract the ID. This means that for example, in the file "12334_ABC.txt" the digits before the underscore state our identifier and the characters after the underscore refer to the initials of the person evaluating the sequence. As already mentioned, this identifier needs to be numeric for tumour or association studies, but not necessarily for population data. The advantage of using the filename as an identifier is that it simplifies the ability to edit ID typing errors. Furthermore, the file extension of the mutation reports represents the corresponding input format (".sqs" for SeqScape, ".txt" for Sequencher) (Figure 3).

### Export of mtDNA

**Figure 4:**
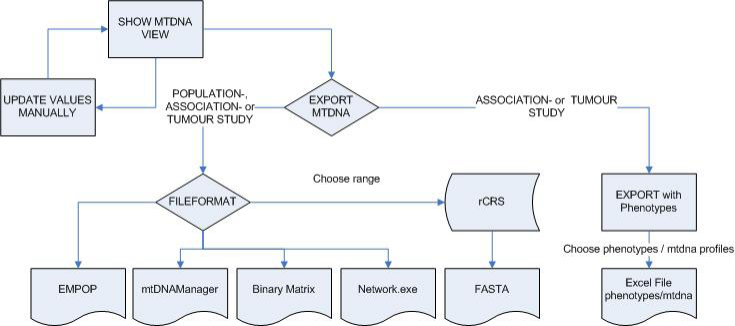
**Flowchart of the mtDNA export process**. mtDNA data can be modified (where each modification is stored by eCOMPAGT's history function) and exported. If the project is a gene-phenotype association study or a tumour study, the export with phenotypes is enabled. Otherwise the user can pick out from several file formats to proceed with further downstream data analysis.

The mtDNA extension of eCOMPAGT can generate several different file export formats for further downstream analysis of mtDNA data. For that purpose, we divide the exports into two classes: export of mtDNA only and mtDNA data complemented by corresponding phenotypes. This is where the actual strength of eCOMPAGT comes into play (Figure 4) with the design of two different graphical user interfaces:

The first GUI consists of two tables, one for mtDNA data only, which displays the imported polymorphisms for each sample, as well as a binary matrix (see below). The imported data can be modified and each modification is logged by eCOMPAGT's history function. A history function (Table 1) enables an easy and reliable tracking of data modifications. It provides information on the person who modified data (employee), on the time-point when data were modified (date & time), on the kind of modified data (location), and on the modified value (old value and new value).

**Table 1:** Example file of eCOMPAGT's history function

Project	Employee	Date	Location	Old value	New value
Burma1	Sebastian	2009-10-09 15:07	Export XLS:\home\sebastian\1.xls	1C	11C
PopTest	Hansi	2009-10-17 16:12	MTDNA haplogr.: Sample17	null	F1a1c
Burma2	Anita	2009-10-17 17:43	MTDNA haplogr.: Sample17	F1a1c	F1a1
Burma2	Florian	2009-10-17 19:11	MTDNA haplogr.: Sample17	F1a1	
PopTest	Gunther	2009-10-18 09:31	Deleted: Phenotype BMI		
...	...	...	...	...	...

• Export of the binary matrix as a tab-delimited file: this file can be opened in any spreadsheet application or text editor; all observed polymorphisms are listed in the header row, the sample names are listed in the first column; the cell entries corresponding to each sample are either 1 if the sample has the specific polymorphism headed in the given column or 0 otherwise.

• EMPOP file format: sample identifier (sample ID), haplogroup, frequency and the profiles as differences to the rCRS are indicated per row for each sample. If the haplogroup is unknown a question mark is inserted instead.

• Tab-delimited file for spreadsheets (MS Excel, Open Office): like the EMPOP file format with the additional columns "range" and "tumour profiles".

• mtDNAmanager: adapted to be imported in mtDNAmanager, this file format fulfils additional grouping of mtDNA data as an extension to the EMPOP format. The three ranges of grouping are: [16024-16569], [1-437], [438-576].

• Network.exe: this file format displays polymorphisms in the first row and lists the mtDNA as a sequence, in which the reference nucleotide is indicated in case of absence of a given polymorphism in the corresponding sample. The sample names can be up to 13 characters long. If insertions of 3 or more nucleotides appear, the Fluxus Network application gives a warning stating duplicated header information.

• FASTA file format: the user can choose any ranges, single positions or any arbitrary combination of ranges which results in a file of DNA sequences for each sample.

The second GUI allows the connection of mtDNA data to phenotypes as required in association or tumour studies. Two adjacent tables show phenotypes listed row by row in the left hand table and mtDNA polymorphisms in the right hand table as a list of all mtDNA mutations. This enables the user (assuming that he has the corresponding privileges to export data) to select either all phenotypes and mtDNA polymorphisms or any combination of arbitrary phenotypes and mtDNA data. This file is exported as a standard BIFF8 format (MS Excel, Open Office) and can be further analyzed with statistics applications.

### Sample information

Storage of sample information in a database system is a highly sensitive issue, and it is one of the most important design features to restrict access to this information. A software based authorization system is not sufficient in such a case, which means that the database itself needs to authorize the access. eCOMPAGT achieves this by creating database views, separate database user accounts and decides if a user is allowed to access all sample data according to internal access rights. To let the system know which data is actually sample relevant, it has to be selected initially at the phenotype import step, in which the columns containing sample specific information are also included. The selected sample columns are then stored in a separate database relation, accessible only with special database access rights.

## Further features

### Support of MySQL and Oracle database systems

eCOMPAGT can be run under MySQL and Oracle. The support of both systems should lead to a higher user accessibility and make the set up for small laboratories much easier through the use of MySQL as background database system.

### Enhancement of other genotyping methods

eCOMPAGT's new release enables the import and storage of STR (Short Tandem Repeats) data exported from GeneMapper (Applied Biosystems, Foster City, USA). The import of phenotypes is possible at any time, as for Taqman and SNPlex data. In addition, besides the storage and the combined export of SNPlex data with corresponding phenotypes, we enhanced eCOMPAGT to enable quality control statistics. This feature is useful in the laboratory, where double genotyping is performed and positive controls need to be checked. This data can be exported and viewed in spreadsheet applications, which provide the scientists with a powerful tool for easy error recognition. Before a final export (combination of phenotypes and related genotypes) can be executed, the double entries of samples need to be deleted. The elimination of double entries is logged by eCOMPAGT and is only executable by a user with dedicated rights (defined by eCOMPAGT).

## Utility and Discussion

### Import from various sources

eCOMPAGT presents the possibility to analyse mtDNA profiles generated by two independent sequence evaluators in a single step, each of them possibly using different computer programs (Sequencher, SeqScape). The systems export formats are recognized automatically, the files are imported into eCOMPAGT and are then compared in a pairwise manner. This feature reflects an important validation process for quality management, since errors in the evaluation process and different interpretations of e.g. point or length heteroplasmy can be detected. Additionally, transcription errors can be avoided through the automated import process.

### Export to various software programs and population databases

In forensic genetics it is necessary to determine the rarity of a given mtDNA profile. Therefore databases like EMPOP [17,18] or mtDNAmanager [19] exist. Our software generates export formats for both databases. Furthermore, mtDNA data can be saved as network.exe file format, otherwise a complex and error prone task. For phylogenetic programs like PAUP*, PHYLIP, MrBayes, Migrate, Arlequin etc. we created the FASTA file, a format which can then be easily adapted to each particular program For association studies, which aim at identifying genetic markers that are associated with certain phenotypes, we created a binary matrix. This binary matrix can be imported directly into statistical programs like SPSS, SAS or R, where association studies are executed primarily. The storage of haplogroups allows further quality controls (e.g. correct interpretation of length heteroplasmies) and association studies (e.g. affinity of specific haplogroups to a disease).

### Comparison to other systems

The software mtDNAmanager allows the storage of mtDNA sequence data, gives an estimate of the most-probable mtDNA haplogroup affiliation and provides frequency estimates on the rarity of mtDNA profiles. Therefore, mtDNAmanager can be used as downstream application to eCOMPAGT. In a similar manner, EMPOP can also be used for frequency estimates and for the construction of quasi median networks [21].

### New features compared to the previous version of eCOMPAGT

The new version of eCOMPAGT was extended to various applications of mtDNA genotyping:

• Storage and management of mtDNA data for population genetics, tumour dynamics and genotype-phenotype association studies;

• Validation process for mtDNA profiling (by importing and comparing mutation reports generated by SeqScape and Sequencher);

• Export of mtDNA profiles in EMPOP [17,18] format, as a binary matrix, as tab-delimited files for Excel, as mtDNAmanager [19] format, as input file for Network.exe [20], and as FASTA format.

Further enhancements of eCOMPAGT include:

• Import, storage and management of STR-data;

• Direct support of MySQL;

• Check of quality controls (double genotyping) for SNPlex.

### Strength and limitations

The key features of our software are summarized in Table 2. Our software has several strengths: (1) with eCOMPAGT we provide automatic import and validation of mtDNA profiles combined with various export formats. Many error prone and time consuming tasks can therefore be avoided or simplified; (2) the usage of the freely available MySQL database allows the installation and use of our software with no additional licence costs. Furthermore, all user-related information is securely stored in the database, supported by separated database accounts and usage of database views; (3) classical elements of a customer relationship management, combined with data management for numerous genotyping methods and the linkage to phenotypes render eCOMPAGT a powerful tool which covers different aspects of the laboratory workflow.

**Table 2:** Key features of the mtDNA-version of eCOMPAGT

Advantages	Disadvantages
Import of population data in EMPOP format	No automatic haplogroup assignment
Import of Sequencher/SeqScape mutation reports	
Upload of phenotypes at any time	
Comparison of double evaluations	
History function for traceability	
MySQL and ORACLE support	
Export of several file formats:	
FASTA	
Network.exe	
mtdnaManager	
EMPOP	
binary matrix as a tab-delimited file	
Excel format with phenotypes	
Tab-delimited file for spreadsheets	
Tumor mutations: Automatic detection of somatic mutations	

Our software has limitations as well, as eCOMPAGT doesn't automatically assign mtDNA haplogroups to samples. However, the assignment of haplogroups to mtDNA profiles is difficult, especially if only control region information is available, and most haplogroups are defined by coding region polymorphisms [22]. Therefore, instead of tempting the user to trust incomplete or inconsistent haplogroup assignments, we decided to exclude this function from eCOMPAGT and to give the user the opportunity to enter, store, review and update the haplogroup affiliation of the samples.

## Conclusions

eCOMPAGT has been enhanced by the import, storage and management of mtDNA profiles. This makes the software interesting not only for epidemiologic laboratories, but also for forensic institutes, human-genetic facilities and anthropological research laboratories, which are engaged with mtDNA in terms of population genetics and phylogenetics. Errors in mtDNA profiles, which led to erroneous conclusions and endless discussions in recent years, can therefore be avoided. Furthermore, the field of genetic epidemiology is expanded by mitochondrial association studies.

## Availability and Requirements

Project name: eCOMPAGT

Project home page: http://dbis-informatik.uibk.ac.at/ecompagt

Operating system(s): Platform independent

Programming language: Java

Other requirements: Java 1.6., relational database (available for Oracle and MySQL, tested for IBM DB2)

## Authors' contributions

SS and HW were responsible for programming and designing eCOMPAGT and drafted the manuscript. AB initialized the project, supervised it and drafted the manuscript. FK and GS drafted the manuscript. All authors read and approved the final manuscript.

## Authors' informations

SS and HW are PhD-students in computer science. AB is a junior group leader in bioinformatics and molecular biology. FK is a full professor for Genetic Epidemiology and GS is a full professor for computer science in the area of databases and information systems.
